# Development and initial validation of the Career Self-Management scale for Chinese coaches

**DOI:** 10.3389/fpsyg.2023.1160584

**Published:** 2023-08-15

**Authors:** Chonghui Zhang, Jing-Dong Liu

**Affiliations:** ^1^School of Physical Education, Chongqing University, Chongqing, China; ^2^Department of Physical Education, Sun Yat-sen University, Guangzhou, China

**Keywords:** career development, Chinese coaches, validity, reliability, invariance analysis

## Abstract

**Objective:**

The purpose of the study was to develop and initially validate a context-specific scale assessing Career Self-Management for Chinese coaches (Career Self-Management Scale-CC; CSMS-CC).

**Methods:**

Firstly, qualitative data obtained from in-depth interview with coaches were contently analyzed to generate potential CSMS-CC items. The content validity of the items was evaluated by a panel of experts. Secondly, the factor structure and item performance of the CSMS-CC were examined using exploratory factor analysis (EFA) and internal consistency reliability of its subscales were evaluated in sample 1 (*n* = 229, 24.01% females). Thirdly, factor structure of the CSMS-CC was further examined using confirmatory factor analysis (CFA) in sample 2 (*n* = 295, 32.54% females). Internal consistency reliability was evaluated using Cronbach’ alpha coefficient and composite reliability. Nomological validity was examined using Pearson correlation and structural equation modeling (SEM) by investigating the correlations between CSMS-CC subscales with career success. Finally, measurement invariance and latent mean difference of the CSMS-CC was examined across gender, professional title and coaching class using multiple-group CFA (MGCFA).

**Results:**

Based on the results of the content analysis and content validity evaluation, 18 CSMS-CC items were retained for further analysis. Results of EFA in sample 1 revealed that eight items were problematic and removed. The second round of EFA revealed that three components were retained and labelled as Networking Behavior (4 items), Training Exploration (3 items), and Guanxi Development (3 items). Results of CFA in sample 2 suggested that the 10-item three-correlated-factors model of CSMS-CC demonstrated acceptable model fit to the data, χ^2^ = 135.01, df = 32, *p* < 0.01, CFI = 0.91, TLI = 0.90, SRMR = 0.05, RMSEA = 0.092 (90% CI = 0.076–0.108). Composite reliability (ranging from 0.84 to 0.88) and Cronbach’s alpha coefficients (ranging from 0.78 to 0.81) of three subscales were found satisfactory. Nomological validity was supported by the results that total score and subscale scores of the CSMS-CC were significantly associated with internal marketability and external marketability. It was found that the CSMS-CC measurement model was strict invariant across gender, professional title and coaching class. Significant differences on all three subscales across professional title and on Guanxi development across coaching class were revealed.

**Conclusion:**

Results of this study provided initial support for the psychometric properties of the 10-item CSMS-CC, which suggested that the CSMS-CC could be used for measuring the career self-management of Chinese coaches.

## Introduction

Career management is a dynamic process that aims to meet the needs of both individuals and their organizations. Both parties should be responsible for the career management of the individuals. The responsibilities of individuals for their career management have attracted extensive attention recently (e.g., Lent and Brown, 2013; Nikandrou and Galanaki, 2016; Elodie and Michael, 2022). Career self-management (CSM), an important concept in industrial and organizational (I/O) psychology with the emphasis on individuals’ personal agency in advancing their own career goals, has gradually became the core of their career management (Greenhaus et al., 2010). CSM was considered important for individuals to attain desired career outcomes in various contexts and for different vocations, including the coaches in sport context. However, the investigation on the CSM among sports coach population has been scarce and deserves more attention from researchers and practitioners. Researchers in I/O psychology field have proposed different conceptual frameworks of CSM, which will inform future works on the CSM among coaches in sport context. For example, Crites (1969) developed a CSM framework of vocational adjustment and described the process of implementing a decision to enter an occupation, adjusting effectively, enriching oneself and progressing within that occupation (p. 355). Early CSM frameworks are concerned with the behaviors that people deployed to advance and develop their careers (Hall, 1990) and to what extent that they integrated these behaviors into the process of career development (Stumpf et al., 1983; Gould and Penley, 1984). Sturges et al. (2002) treated networking behaviors, visibility behaviors, and mobility-oriented behaviors as CSM behaviors in their research and tended to consider CSM behaviors as a kind of vocational behaviors that people performed throughout the course of their working lives. King (2004) considered CSM as a dynamic process that involves the execution of a set of co-occurring behaviors including positioning behaviors, influence behaviors, and boundary management. The core assumption of King’s CSM framework is that individuals cannot always decide how their careers would develop because they are dependent on the interventions provided by gatekeepers and the contextual factors. King’s (2004) definition of the CSM has been widely accepted and used in previous research (Sanders et al., 2022; Retkowsky et al., 2023), which will inform future research on coaches’ CSM by treating it as a process including behaviors and strategies coaches taken to realize their career development and advancement.

Extending previous theories, some other researchers explicitly integrated specific or general goals into the process and considered CSM behaviors as the strategies that individuals use to achieve their career goals, which may involve conscious choices and activities designed to help a person achieve his/her career goals. For example, Lord et al. (2010) conceived people as active agents of their own development and were capable to create and set goals, monitor available supports and constraints for goal attainment, translate goals into action plans, execute plans via diverse behaviors, monitor actions and outcomes, and process feedback to change goal, action plans, and behaviors accordingly. Similarly, Greenhaus et al. (2010) defined CSM as a process by which individuals develop, implement, and monitor career goals and strategies. Seibert et al. (2013) developed a CSM model including intrinsic career goals, extrinsic career goals, career planning, and career satisfaction. It is a kind of proactive regulation to develop career strategies, goals and plan. Lent and Brown (2013) considered CSM as a process that individuals use to stay focused, plan and coordinate actions, and persist when things get difficult in the context, with the emphasis on individuals’ proactivity and self-regulation. Thus, the individuals can develop a range of behaviors to control his/her career. Spurk et al. (2018) treated CSM as a process of intentionally building, maintaining, and using various personal and contextual resources such as goal setting, mapping the environment for resources, planning, monitoring actions, and feedback processing that lead to positive career outcomes. There is also a European model developed by Pinto and Taveira (2010) which includes four behaviors, namely, career exploration, goal setting, design and implementation of action plans, and monitoring and feedback obtaining. However, it is noteworthy that all CSM processes abovementioned are consisted of different kind of behaviors or strategies rather than attitudes, abilities or other psychological aspects that individuals use to advance or develop their careers. These behaviors are adaptive career behaviors related to but different from career adaptability proposed by Savickas (1997), which is defined as the readiness to cope with the predictable tasks of preparing for and participating in the work role and with the unpredictable adjustments prompted by change in work and working conditions (p. 254). Table 1 presents a summary on the CSM dimensions proposed in previous research.

**TABLE 1:** Summarized structures and dimensions of career self-management.

References	Dimensions	Dimensions and content
Stumpf et al., 1983	3	The exploration process, reaction to exploratory behavior, and beliefs about exploratory behavior
Gould and Penley, 1984	7	Seeking guidance/mentoring, networking, self-presentation, creating opportunities, extended work involvement, other enhancement, and opinion conformity
Pazy, 1988	3	Career planning, career tactics, and proactivity
Kossek et al., 1998	4	Developmental feedback seeking, job mobility preparedness, career training, and career self-efficacy
Greenhaus et al., 2010	5	Career goals, the development and implementation of career plans and strategies, and feedback
Long et al., 2002	5	Exploration, career goal setting and strategy identification, continuous learning, self-nomination, and interpersonal relationship orientation
Sturges et al., 2002	3	Networking behaviors, visibility behaviors, and mobility oriented behaviors
King, 2004	3	Positioning behaviors, influence behaviors, and boundary management
Ling and Ou, 2010	5	Know development opportunity of organization, career belief, career exploration, self-cognition, and upward communication
Seibert et al., 2013	4	Intrinsic career goals, extrinsic career goals, career planning, and career satisfaction
Nikandrou and Galanaki, 2016	4	Task-oriented strategies: extended work involvement and maintain career flexibility; relations-oriented strategies: self-presentation and seeking mentoring
Spurk et al., 2018	5	Goal setting, mapping the environment for resources, planning, monitoring actions, and feedback-processing

Most of previous research were conducted in Western cultures and therefore it is critical to delineate which types of behaviors may best represent the CSM structures in Chinese culture. According to King (2004), CSM behaviors are often protean and used to achieve career success, which could be reflected and predicted by marketability and perceptions of fit (Haines et al., 2014; Park et al., 2022). Elodie and Michael (2022) advocated that researchers should focus on specific CSM behaviors and how these behaviors can help individuals direct their own career development and manage career changes in their social-culture. Therefore, it is important to pay attention to the reciprocal interplay of personal and contextual factors that would influence individuals’ purposive career behaviors (King, 2004; Lent and Brown, 2013; Hodgson et al., 2017; Holmes et al., 2021). Makkonen (2016) studied CSM of Western expatriates working in organizations in China and observed individuals’ changed career expectations and employment context of the host country. Previous research has reported distinctive characteristics of the CSM in Chinese context that derived from the indigenized social-culture in China. For example, new dimensions of interpersonal interaction orientation and upward communication emerged from studies by Long et al. (2002) and Ling and Ou (2010) respectively in Chinese culture, which were not reported previously in Western culture.

Coach, as a specialized vocation, can be inherently stressful. Coaches may encounter a variety of organizational, contextual, interpersonal, and intrapersonal stressors and need to fulfill multiple roles and undertake various responsibilities, which may require them to develop their own ways to successfully manage their career. Werthner and Trudel (2009) investigated the idiosyncratic learning paths taken by Canadian coaches in the development of coach expertise and found that persistent learning behaviors were perceived to be valuable and had long-term impacts on their career development. For example, being an active learner and deciding to engage in frequent learning experiences would be beneficial for coaches’ development and advancement, which would affect their development in coaching knowledge, skills, and abilities (Mallett et al., 2016; Holmes et al., 2021). Being a lifelong learner was considered as a prerequisite for becoming a skilled coach as the experiences accumulated from their unique learning paths were important for their career development (Werthner and Trudel, 2009). Moreover, Chinese coaches may encounter unique situational factors that derived from the Whole Nation System in sport in Mainland China, which is contingent on the structure of the country creating and implementing sport-related policy (Si et al., 2015). For Chinese coaches, they may unavoidably develop their unique ways to manage their career. Therefore, for research on CSM among Chinese coaches in sport context, not only cultural factors but also situational factors should be taken into consideration. It is imperative to establish a CSM framework that is suitable for Chinese coaches in sport context (Zhang and Ma, 2009). Meanwhile, the development of a psychometric measure that could be used to assess CSM for Chinese coaches would advance the research on the topic.

Collectively, to shed light on the abovementioned limitations, the purpose of the study was to develop and initially validate a context-specific scale assessing career self-management of Chinese coaches (Career Self-Management Scale for Chinese coaches; CSMS-CC). Firstly, potential CSMS-CC items were developed based on previous literatures and qualitative content analysis of the interviews with coaches. Content validity was examined by inviting a panel of experts to evaluate to what extent that the items capture the concept of CSM. Secondly, the factor structure and item performance of the CSMS-CC were examined using exploratory factor analysis (EFA) in sample 1. Thirdly, factor structure and nomological validity were further examined in an independent sample. Finally, measurement invariance and latent mean differences of the CSMS was examined across gender, professional title, and coach class.

## Materials and methods

### Item development and content validity

An initial item pool was developed based on literatures. Guidelines for item wording were closely followed to maximize the clarity, specificity, and shortness of items (DeVellis, 1991). These items were used for discussion with coaches during subsequent interviews. A sample 16 coaches (11 males and 5 females) ranging in age from 35 to 56 years old (*M* = 47; SD = 8.69) was invited to participate in one to one in-depth interview. The coaches were from five sports including badminton (*n* = 2), swimming (*n* = 3), gymnastics (*n* = 4), diving (*n* = 3), and fencing (*n* = 4). Their coaching experience ranged from 5 to 32 years (*M* = 21.25; SD = 7.64). Their professional titles are national (*n* = 8) and international coaches (*n* = 8). An in-depth interview protocol was developed to facilitate the interview and discussion relating to their behaviors in the process of CSM. The interviews lasted approximately 40 min ranging from 30 to 60 min. During the interview, the coaches were asked to suggest for item refinements and potential alternative items. Interview transcripts were qualitatively content analyzed (Côté et al., 1993) based on the definitions of CSM in literatures (Long et al., 2002; Zhang and Ma, 2009; Ling and Ou, 2010; Holmes et al., 2021). Initially, a total of 24 items belonging to four dimensions (Networking Behavior: 5 items; Training Exploration: 8 items; Career Goal and Strategy: 5 items; and Guangxi Development: 6 items) were obtained throughout the abovementioned process (see Table 2).

**TABLE 2:** Content validity index of items.

Items	CVIs	Dimensions
A1: I often attend symposiums on coaching so that I can communicate and get in touch with master coaches.	0.9	Networking behavior
A2: I try to coach in an innovative way based on the training principle.	0.8	Training exploration
A3: I often reflect whether my current training approach is reasonable or not.	0.9	Training exploration
A4: I actively pursue and exert my potential to make me in an ideal professional state.	0.8	Training exploration
A5: I have a career planning for myself.	0.8	Career goal and strategy
A6: I have a clear vision about my professional development.	0.9	Career goal and strategy
A7: I have my ways and strategies to achieve my career goals.	0.9	Career goal and strategy
A8: I have short-term and long-term career goals.	0.9	Career goal and strategy
A9: I often communicate with colleagues on professional issues.	0.9	Training exploration
A10: My colleagues and me often attend coach development education and workshops.	1.0	Networking behavior
A11: I set professional goals with specific plans to achieve them.	0.9	Career goal and strategy
A12: I take great efforts to develop professional skills.	0.8	Training exploration
A13: I often help others and get support from others in my team.	1.0	Networking behavior
A14: I develop a networking through which I can learn about most update and important information in the field.	1.0	Networking behavior
A15: I get close contact with influential members in our organization.	0.9	Guanxi development
A16: I often report my progress to my supervisor privately.	0.9	Guanxi development
A17: I try to let my supervisor know what I want to do.	1.0	Guanxi development
A18: I often express my professional aspirations and strategies to my supervisor privately.	0.9	Guanxi development
A19: I often attend coach development training programs organized by authoritative figures and organizations.	*0.7*	*Training exploration*
*A20: I am a member of some organizations or associations of coach.*	*0.7*	*Networking behavior*
*A21: I often communicate with sports science experts about my training.*	*0.6*	*Training exploration*
*A22: I often visit some leaders and gatekeepers.*	*0.5*	*Guanxi development*
*A23: I have a special guanxi with the leader in charge or my supervisor.*	*0.4*	*Guanxi development*
*A24: My mentor provides me supervision on my coaching.*	*0.5*	*Training exploration*

Items with CVI values lower than 0.8 in italic were removed from further analysis.

Furthermore, a panel of 10 experienced applied sport psychology experts was invited to evaluate the content validity of the 24 items by indicating to what extent that each item satisfactorily taped the meaning of their correspondent dimensions on a 4-point Likert scale ranging from 1 (poor match) to 4 (excellent match) based on the definitions of the CSM dimensions that obtained from content analysis. Specifically, Networking Behavior dimension measures coaches’ active network building to obtain latest information, gain knowledge and establish good work alliance. Career Goal and Strategy measures progressive strategies that increases the likelihood of career goal attainment. Training Exploration dimension measures behaviors that related to learning, exploring, and developing professional training with changes and innovation. It may include coaches’ learning, the development of coaching expertise, coaching experiences, getting insight from experiences, and have training changes and innovation. Guanxi Development dimension measures those behaviors of coaches such as progress reporting, professional aspiration and strategies expressing to supervisor in private. The Content Validity Index (CVI; Lynn, 1986) was calculated for each item to inform final decisions on whether the items should be retained, eliminated, or revised. The experts were also asked to propose alternative items and advise for improving items if needed. The CVI of each item was calculated by dividing the number of experts who gave a rating of 3 or 4 on specific item. Items with CVIs larger than 0.80 would be retained (Polit et al., 2007). Six items with CVIs less than 0.80 were deleted. In addition, minor revisions were made on specific items based on feedbacks from experts to make items more accurate and clearer. Finally, 18 items representing four dimensions (Networking Behavior: 4 items; Training Exploration: 5 items; Career Goal and Strategy: 5 items; and Guangxi Development: 4 items) were retained for subsequent quantitative analysis (see Table 2).

### Participants and procedure

#### Sample 1

A total of 229 coaches (171 males, 55 females, 3 missing) with an average age of 37.44 years old (SD = 8.867) was invited to participate in this study by completing the 18-item CSMS-CC. All data were identified as valid and used for further data analysis. Coaches were from 17 sports including badminton, tennis, trampoline, martial arts, diving, swimming, athletics, table tennis, fencing, weightlifting, free combat, boxing, wrestling, shooting, archery, rowing, and canoeing at four Provincial Training Centers (PTC: Guangdong, Guangxi, Shanghai, and Chongqing). They had an average of 5.05 years (SD = 2.12) coaching experience. Coaching class means coaching position which includes two levels, namely, municipal level and provincial/national level. Sixty-three coaches were municipal level and 166 coaches were provincial/national level at the moment of data collection. Professional title refers to the professional qualification level, which includes three levels, namely, junior, middle, and senior levels. One hundred and thirty-five coaches were middle level and 94 coaches were senior level at the moment of data collection.

#### Sample 2

A total of 295 coaches (199 males and 96 females) with an average age of 37.31 years old (SD = 8.677) was invited to participate in this study by completing questionnaires measuring CSM and career success. All data were identified as valid and used for further data analysis. Coaches were from 16 sports including race walking, cycling, gymnastics, judo, taekwondo, rifle, windsurfing, acrobatic skills, sailing, modern pentathlon, decathlon, flying saucer, discus, javelin, boxing, and swimming at five PTCs (Shandong, Jiangsu, Hebei, Hubei, and Zhejiang), with an average of 4.1 years (SD = 1.94) coaching experience. Seventy-nine coaches were municipal level and 216 coaches were provincial/national level. Regarding professional title, 184 coaches were middle level and 111 coaches were senior level at the moment of data collection.

#### Data collection

Coaches from nine PTCs in China were contacted and invited to participate in this study. Informed consent was distributed and obtained before data collection and only participants who returned their consent form were asked to complete questionnaires. Data were collected between January and November, 2021. Instructions of survey were clearly stated at the beginning of the survey. All participants took part in the study voluntarily and anonymously.

### Measures

#### Career self-management

The initially developed Chinese version of the CSMS-CC includes 18 items measuring four dimensions. Participants were asked to indicate to what extent that they agreed or disagreed with each statement on a 4-point Likert scale, ranging from 1 (Strongly disagree) to 4 (Strongly agree) based on their general experiences throughout their careers.

#### Career success

The Chinese version of the Internal and External Marketability scale (Yan et al., 2008) was used to assess career success, which measures perceptions of marketability within one’s organization and perceptions of marketability in the external marketplace (Eby et al., 2003). The scale includes 6 items with 3 measuring Internal Marketability (example item “My institute views me as an asset to the organization”) and External Marketability (example item “Given my skills and experience, other organizations view me as a value-added resource”). Participants were asked to respond on a 4-point Likert scale ranging from 1 (strongly disagree) to 4 (strongly agree). Previous research has demonstrated that the scale displayed satisfactory validity and reliability among Chinese population (Yan et al., 2008). Internal consistency reliabilities of the two subscales were 0.756 and 0.758, respectively in this study. Marketability is an important indicator of career success, has been considered as one of the consequent variables of CSM. Therefore, the relationship between CSMS-CC and career success was examined to evaluate the nomological validity of the CSMS-CC.

### Data analysis

SPSS (Version 23.0. Armonk, NY, USA: IBM Corp.) and Mplus (Muthén & Muthén, 1998–2014) software were used for data analysis. Firstly, EFA using the principal axes factoring method with Promax oblique rotation was carried out to explore the factor structure of the CSMS-CC in sample 1. The factor extraction was based on the eigenvalue value (greater than 1.0). Criteria for filtering items included communalities that were less than 0.50, item loading that was less than 0.50 on any factor and with 0.40 or higher cross loadings on multiple factors (Pan et al., 2017). Internal consistency reliability was evaluated using Cronbach’s alpha. Secondly, confirmatory factor analysis (CFA) based on the robust maximum likelihood (MLR) estimator was conducted to evaluate the factor structure of the CSMS-CC (Finney and DiStefano, 2006). The adequacy of the model to the data were evaluated using multiple fit indices, such as the Chi-square statistic (χ^2^), the Comparative Fit Index (CFI), the Tucker Lewis Index (TLI), the Root Mean Square Error of Approximation (RMSEA) and Standardized Root Mean Square Residual (SRMR). Although values indicative of acceptable model fit remain controversial, it is typically accepted that an acceptable fit is indicated by values of 0.90 and above for the CFI and TLI and for the RMSEA and SRMR, values of 0.08 and 0.06 or less, respectively (Hu and Bentler, 1999). Composite reliability and average variance extracted (AVE) were calculated to evaluate the reliability of the subscales. Thirdly, nomological validity was evaluated by examining the correlations of total score and subscale scores of the CSMS-CC with internal marketability and external marketability using Pearson correlation and structural equation modeling (SEM). Finally, multiple-group CFA was conducted to examine the measurement invariance and latent mean difference of the CSMS-CC across gender, professional title, and coaching class. Specifically, a baseline model was established and then four increasingly more constrained models were specified to examine the configural, metric, scalar, and residual invariance. Once metric and scalar invariance was evidenced, latent mean difference was conducted across different groups. For measurement invariance evaluation, as the Chi-square difference test is sample size sensitive, the differences in the descriptive fit indices (ΔCFI and ΔRMSEA) were used in model comparisons in this study. According to Chen (2007), non-invariance is indicated by a change of ≥0.010 in the CFI supplemented by a change of ≥0.015 in the RMSEA.

## Results

### Results of sample 1

Exploratory factor analysis was advocated during the early stages of scale development to avoid misspecification of the number of factors and to maximize the convergent and discriminant validity of the items constituting each factor. Results of Kaiser-Meyer-Olkin (KMO = 0.933) and Bartlett’s test of sphericity (*p* < 0.001) suggested that the data was appropriate for EFA. Eight items were removed due to high cross factor loadings or low primary factor loadings (less than 0.5). Results of the second round EFA with the remaining 10 items revealed that three components were retained and accounted for 68.57% of the total variances. Primary factor loadings of the ten items were larger than 0.5 ranging from 0.64 to 0.93 and cross factor loadings were less than 0.29. Moderate inter-factor correlations were found ranging from 0.46 to 0.56. Further analysis revealed that the three components could be represented by three components, namely, Networking Behavior (factor 1: 4 items), Training Exploration (factor 2: 3 items), and Guanxi Development (factor 3: 3 items). The Cronbach’s alpha coefficients of the three dimensions ranged from 0.78 to 0.81. Table 3 presents the descriptive statistics and results of the second round EFA.

**TABLE 3:** Descriptive statistics, factor loading and correlation of second round EFA (sample 1, *n* = 229).

Dimensions/items	*M* (SD)	F1	F2	F3
**F1: networking behavior**				
A10	2.90 (0.80)	0.85		
A1	2.86 (0.76)	0.83		
A14	2.73 (0.75)	0.65		
A13	3.01 (0.73)	0.64		
**F2: training exploration**				
A3	3.36 (0.57)		0.91	
A4	3.37 (0.57)		0.80	
A2	3.29 (0.54)		0.76	
**F3: guanxi development**				
A17	2.94 (0.69)			0.93
A16	2.84 (0.69)			0.76
A18	3.07 (0.68)			0.70
68.570% of explained variances		43.427%	12.995%	12.148%
**Factor correlations**	***M* (SD)**	**Cronbach’s alpha**	**F1**	**F2**
F1: networking behavior	11.48 (2.43)	0.78		
F2: training exploration	9.99 (1.45)	0.81	0.53**	
F3: guanxi development	8.90 (1.85)	0.78	0.56**	0.46**

**p < 0.01.

### Results of sample 2

#### Factor structure and internal consistency reliability

Table 4 presents the descriptive statistics of sample 2. Confirmatory factor analysis was conducted to further evaluate the factor structure of the 10-item CSMS-CC that generated in EFA. It was found that the 10-item three-factor model of CSMS-CC demonstrated an acceptable model fit to the data of sample 2, χ^2^ = 135.01, df = 32, *p* < 0.01, CFI = 0.93, TLI = 0.90, SRMR = 0.05, RMSEA = 0.092 (90% CI = 0.076–0.108). Standardized factor loadings ranged from 0.70 to 0.90. Composite reliability of three subscales ranged from 0.84 to 0.88, showing satisfactory reliability. The values of AVE of the three factors were greater than 0.50 (see Table 5). The inter-factor correlations among the three factors ranged from 0.45 to 0.53 (*r*_*networking behavior*–training exploration_ = 0.51; *r*_*networking behavior*–guanxi development_ = 0.53; *r*_*training exploration–guanxi development*_ = 0.45). The Cronbach’s alpha coefficients of the three dimensions were 0.78 (Networking Behavior), 0.79 (Training Exploration), and 0.81 (Guanxi Development), respectively. Comparison on the three-factor model with the one-factor model provided additional support for the three-factor model because the one-factor model exhibited a poor fit to the data, χ^2^ = 292.54.01, df = 35, *p* < 0.01, CFI = 0.77, TLI = 0.70, SRMR = 0.09, RMSEA = 0.161 (90% CI = 0.144–0.179), and the three-factor model outperformed the one-factor model. Collectively, these results suggested that the 10-item three-factor CSMS-CC demonstrated satisfactory validity and reliability.

**TABLE 4:** Descriptive statistics and inter-item correlations (sample 2, *n* = 295).

Item	Skewness	Kurtosis	A13	A14	A10	A1	A2	A3	A4	A16	A17	A18
**Networking behavior**												
A13	-0.580	0.176	1									
A14	-0.163	-0.315	0.555**	1								
A10	-0.404	-0.310	0.442**	0.435**	1							
A1	-0.528	0.250	0.397**	0.395**	0.612**	1						
**Training exploration**												
A2	-0.058	-0.600	0.363**	0.383**	0.380**	0.394**	1					
A3	-0.418	0.513	0.289**	0.315**	0.264**	0.265**	0.555**	1				
A4	-0.384	-0.306	0.384**	0.378**	0.378**	0.391**	0.575**	0.638**	1			
**Guanxi development**												
A16	0.083	-0.300	0.428**	0.362**	0.369**	0.325**	0.256**	0.206**	0.300**	1		
A17	-0.360	0.029	0.325**	0.303**	0.282**	0.226**	0.296**	0.248**	0.333**	0.597**	1	
A18	-0.361	-0.056	0.402**	0.406**	0.339**	0.306**	0.391**	0.381**	0.498**	0.470**	0.601**	1

**p < 0.01.

**TABLE 5:** Standardized factor loading, residual, squared multiple correlation, item-total correlation, Cronbach’s alpha, average variance extracted, and composite reliability of CFA (sample 2, *n* = 295).

Dimensions/items	Loading	Residual	SMC	Item-total *r*	Cronbach’s alpha	AVE	CR
Networking behavior					0.78	0.57	0.84
A14	0.79	0.38	0.62	0.61			
A13	0.77	0.41	0.59	0.58			
A10	0.75	0.43	0.56	0.58			
A1	0.70	0.51	0.49	0.57			
Training exploration					0.79	0.71	0.88
A2	0.83	0.31	0.69	0.57			
A3	0.90	0.19	0.81	0.48			
A4	0.80	0.36	0.64	0.64			
Guanxi development					0.81	0.64	0.84
A16	0.83	0.31	0.69	0.57			
A17	0.71	0.50	0.50	0.51			
A18	0.86	0.26	0.74	0.63			

#### Nomological validity

**FIGURE 1:**
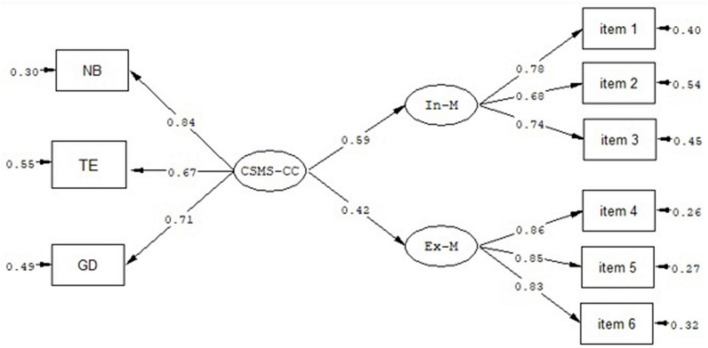
Relationships of the latent construct of the Career Self-Management Scale-Chinese coaches (CSMS-CC) with internal and external marketability. NB, networking behavior; TE, training exploration; GD, Guanxi development; CSMS-CC, Career Self-Management Scale-Chinese coaches; In-M, internal marketability; Ex-M, external marketability.

Nomological validity was evaluated by examining the relationships between scores derived from the three subscales of the CSMS-CC and career success. According to previous findings, marketability is an important criterion of career success (Arthur and Rousseau, 1996; Arthur et al., 2005). It was expected that the subscales of the CSMS-CC would be significantly associated with internal marketability and external marketability. It was found that the correlations of the scores derived from the 10-item CSMS-CC were positively associated with internal marketability and external marketability (see Table 6). In addition, SEM was further performed using Hou et al.’s (2004) parcel strategy to evaluate the relationships between the latent construct of the CSMS-CC and internal marketability and external marketability (see Figure 1). The results of the SEM demonstrated an acceptable model fit to the data, χ^2^ = 58.58, df = 25, *p* < 0.01, CFI = 0.95, TLI = 0.91, SRMR = 0.06, RMSEA = 0.094 (90% CI = 0.074–0.11). This result is consistent with the previous findings (e.g., Noe, 1996; Long and Mao, 2002; Eby et al., 2003) and provides support for the nomological validity of the CSMS-CC.

**TABLE 6:** Correlations of subscale scores and total score of the CSMS-CC with its theoretically related variables (sample 2, *n* = 295).

CSMS-CC	Career success total score	Internal marketability	External marketability
Networking behavior	0.386**	0.411**	0.244**
Training exploration	0.301**	0.202*	0.283**
Guanxi development	0.356**	0.358**	0.243**
Total CSMS-CC score	0.426**	0.407**	0.307**

*p < 0.05, **p < 0.01.

#### Invariance analysis

Table 7 presents the goodness-of-fit indices for the invariance models. Results of comparisons between the more and less constrained models across samples showed no significant changes in CFI (ΔCFI) and RMSEA (ΔRMSEA) exceeded the recommended cut-off value (0.01 and 0.015). These results suggested that the measurement model of the CSMS-CC was strict invariant across gender, professional title, and coaching class, which provided additional support for the psychometric properties of the 10-item three-factor CSMS-CC.

**TABLE 7:** Results of invariance analysis (sample 2, *n* = 295).

Model	χ^2^	df	CFI	TLI	RMSEA (90% CI)	SRMR	ΔCFI	ΔRMSEA
**Gender (male = 199; female = 96)**								
Configural	160.224	64	0.896	0.854	0.101 (0.082–0.121)	0.063	–	–
Metric (weak invariance)	167.443	71	0.896	0.868	0.096 (0.077–0.115)	0.08	0.000	0.005
Scalar (strong invariance)	174.157	78	0.896	0.88	0.091 (0.073–0.110)	0.08	0.000	0.005
Residual (strict invariance)	181.228	88	0.899	0.897	0.085 (0.067–0.102)	0.089	0.003	0.006
**Professional title (middle = 184; senior = 111)**								
Configural	171.484	64	0.884	0.83	0.107 (0.088–0.126)	0.06	–	–
Metric (weak invariance)	168.185	71	0.893	0.864	0.096 (0.078–0.115)	0.067	0.009	0.011
Scalar (strong invariance)	173.978	78	0.894	0.878	0.091 (0.073–0.110)	0.068	0.001	0.005
Residual (strict invariance)	182.679	88	0.896	0.893	0.085 (0.068–0.103)	0.084	0.002	0.006
**Coaching class (municipal = 79; provincial and national = 216)**								
Configural	165.336	64	0.886	0.84	0.104 (0.084–0.123)	0.066	–	–
Metric (weak invariance)	180.457	71	0.877	0.844	0.102 (0.084–0.121)	0.091	0.009	0.002
Scalar (strong invariance)	189.931	78	0.874	0.855	0.099 (0.081–0.117)	0.093	0.003	0.003
Residual (strict invariance)	198.052	88	0.876	0.873	0.092 (0.075–0.109)	0.115	0.002	0.007

χ^2^, the Chi-square statistic; df, degree of freedom; CFI, the Comparative Fit Index; TLI, the Tucker Lewis Index; RMSEA, the Root Mean Square Error of Approximation; 90% CI, 90% confidence interval; SRMR, Standardized Root Mean Square Residual; ΔCFI, change in CFI; ΔRMSEA, change in RMSEA.

#### Latent mean differences

Table 8 presents results of latent mean differences analysis across gender, professional title, and coaching class. There were no significant differences on all three subscales between male and female coaches. Significant differences on all three subscales between different professional titles were revealed with coaches at middle level reporting much higher latent means than their counterparts at senior level. For coaching class, significant difference on guanxi development was observed with coaches from municipal teams reporting much higher latent mean than their counterparts from provincial and national teams.

**TABLE 8:** Latent mean differences between gender, professional title, and coach class (sample 2, *n* = 295).

CSMS-CC	Gender			Professional title			Coaching class		
	Estimate	SE	Estimate/SE	Estimate	SE	Estimate/SE	Estimate	SE	Estimate/SE
Networking behaviors	0.079	0.117	0.668	−0.429	0.115	−3.744**	0.091	0.123	0.743
Training exploration	−0.003	0.119	−0.024	−0.250	0.115	−2.176*	0.009	0.132	0.065
Guanxi development	−0.014	0.084	−0.164	−0.254	0.083	−3.049**	0.220	0.085	2.59**

Groups of coaches who are male, at middle level and training at provincial and national level were treated as reference group respectively in latent mean difference analysis. *p < 0.05, **p < 0.01.

## Discussion

The lack of valid measures of CSM for Chinese coaches has hampered the research on the topic in this population in Chinese context. The present study outlines the process of the development and initial validation of the Career Self-Management Scale, which was specifically designed to measure CSM behaviors and strategies that Chinese coaches taken to achieve their career goals. Collectively, results of the present study indicated that the 10-item three-factor CSMS-CC was a valid and reliable measure that could be used for assessing the CSM of Chinese coaches in Mainland China.

This study opens a new avenue of research on CSM of Chinese coaches in Mainland China. The development of the CSMS-CC makes it possible for researchers and practitioners in future to directly measure and compare the level of career self-managing behaviors of Chinese coaches and further investigate its relationships with antecedent (e.g., personality, motivation, self-efficacy, and career anchors) and consequent (e.g., career and life satisfaction, performance, career success, competitiveness, marketability, and learned helplessness) variables (e.g., Guthrie et al., 1998; King, 2004). The three dimensions of the CSMS-CC emerged from both previous CSM constructs and empirical data in this study ensured the applicability of the CSMS-CC in culture- and domain-specific setting, the Whole Nation System of sport in China (Long et al., 2002). This preliminary scale was reduced to a 10-item scale that measures three dimensions, namely, networking behaviors, training exploration, and guanxi development. The fourth dimension of career goal and strategy was deleted because the five items of the fourth dimension displayed high cross-loadings on other dimensions significantly in the EFA. High item cross-loading reflects that the item may not accurately represent a dimension, which would confound the factor structure of a psychometric instrument. Therefore, the dimension of career goal and strategy was removed from the scale. Some researchers considered CSM behaviors as the strategies that individuals use to achieve their career goals. Following the same logic, in this study, the three retained dimensions refer to an individual’s efforts to realize his/her personal career objectives.

It is worthy to note that the two CSMS-CC dimensions of Networking Behavior and Guanxi Development were purposely designed to accurately capture the salient nature of the Whole Nation System of sport and Chinese culture. Networking Behavior dimension shares common elements with the factor of Interpersonal Relationship Orientation in the CSM construct proposed by Long et al. (2002) in organizational setting in Chinese context, which aims at cultivating influential contacts at work and working alliance. Networking is a job-search behavior and career management strategy (Forret, 2018). Rynne (2012) argued that those retired elite athletes who chose to continue their careers as a coach were also on a high probable career trajectory to securing a high-performance coaching job. This phenomenon was also widely observed among coaches who were elite athletes before their retirement in Chinese context. The experiences of being elite athletes facilitate their networks building when they became coaches, which is much helpful for their career development. Different levels of social networks can open more doors for opportunities. According to King (2004), active network development offers individuals instrumental benefits, such as information, career guidance, and advocacy for promotion or employment. Establishing an external network of personal ties, for example with other members of professional associations, or informal social acquaintances, provides opportunities for interacting with influential members in other organizations, and builds work alliances. The resources obtained from knowing or being known by others, or occupying a position in a social network, have been referred as social capital (Seibert et al., 2001). Those networking behaviors employed by Chinese coaches are efficient means for them to strengthen their social capital. Therefore, the Networking Behaviors items of the CSMS-CC capture coaches’ behaviors related to learning activities, obtaining latest information, and establishing good work alliance. Guanxi Development dimension is similar with the Upward Communication factor from the CSM construct developed by Ling and Ou (2010). Tsui and Farh (1997) defined guanxi as “the existence of direct particularistic ties between two or more individuals.” Guanxi is communal sharing (for example, in a family, subordinates are expected to show unreserved loyalty and obedience toward their superiors), the building of strong personal obligations based on particularistic ties or sentimental ties between the parties involved. In Chinese society, supervisor-subordinate guanxi (SSG) plays an important role in forming and developing trust in one’s supervisor based on their identity of family membership, in-group relationship and work team or group identity building from both supervisors and subordinates’ perspectives (Law et al., 2000; Han et al., 2012). Chinese supervisors may interact with their subordinates differently based on guanxi (Cheng et al., 2002) and offer more bonus and promotion opportunities for those subordinates who have good rather than poor guanxi with them (Law et al., 2000). These subordinates, in turn, would develop greater trust in their supervisors and report better performance (Lin, 2002). Coach, as a traditional-bureaucratic career, is inevitably affected by decisions made by supervisors in the Whole Nation System of sport in China. Therefore, the items of Guanxi Development measure those behaviors related to progress reporting, professional aspiration and strategies expressing, and ideas sharing in private, through which to develop SSG. Our results revealed that the coaches from municipal teams reported much higher latent mean on Guanxi Development, which could be explained by the abovementioned contextual and cultural characteristics in China. For these coaches, they are prone to pay much more attention on Guanxi Development compared to their counterparts from provincial or national teams because they will benefit more from the their guanxi with their supervisors.

Career self-managing behaviors were considered as career strategies (Gould and Penley, 1984), which could be further classified into two categories, namely, relation-oriented strategies and self/work-oriented strategies (Guthrie et al., 1998). The former one involves working through or with other people whereas the latter one focuses on job tasks or the development of career related skills. Following the same lane, both Guanxi Development and Networking Behavior belong to relation-oriented strategies that relate to the socially situated nature of CSM. These two dimensions play salient roles and might determine the efficiency of success pursuit throughout the career development of Chinese coaches. Effective CSM behaviors need to be highly tailor made according to the demands of the situation. It implies that Chinese coaches highlight the importance of networking and supervisors-subordinate guanxi development in the process of CSM. Training Exploration dimension is more related to work-oriented strategies. Coaches are crucial for athletes not only in skills acquisition and perfection but also in their career development. It is the core competence for coaches to cultivate and coach an ordinary athlete to uncover their potential to excel in sports through deliberate training. Training exploration, as a highly profession related dimension, mainly relates to coaches’ active learning, learnt experiences, and self-reflection and insights, which would lead them to train athletes more efficiently.

### Limitations and future directions

Although results of a series of steps provided initial support for the psychometric properties of the CSMS-CC among two samples of Chinese coaches, scale validation is an on-going process and further development and validation of the scale is needed. In the process of scale development and validation, we integrated theoretical and empirical evidences and employed both qualitative and quantitative methods to ensure the CSMS-CC to be applicable to and suitable for the Whole Nation System of sports in China. However, several limitations should be acknowledged. Firstly, all coaches involved in item development stage were at middle or senior levels and the experiences and opinions of coaches at junior level might be overseen. There may be some differences on experiences of CSM between coaches at junior level and those at middle/senior levels. Luckily that all coaches at middle and senior levels should have gone through the process of career development from junior level to advanced levels, which may ensure their responses to interview questions regarding their CSM process have reflected those experiences they had when they were at junior level. Future researchers may consider including more coaches at junior level to further explore and supplement this missing part. Secondly, we did not employ social desirability scale in the process of the development of the CSMS-CC to exclude potential social desirability bias. However, efforts were made to avoid it by asking participants to answer the questionnaires anonymously and reminding them to respond to the questions honestly based on their true experiences and opinions. Future researchers are encouraged to further examine the validity of the scale especially by including a social desirability scale to exclude bias data. Thirdly, two samples were included in this study, although the sizes of the two samples were sufficient for data analysis from statistical perspective, future researchers are encouraged to enlarge the sample size to further examine the psychometric properties of the CSMS-CC. Moreover, unequal sample distribution for gender, professional tittle and coaching class may influence the results of the measurement invariance and latent mean difference analysis. Future research may further investigate the issues using a larger and even distributed sample. Finally, test-retest reliability was not examined in this study, future research may explore this psychometric property. In addition, longitudinal invariance of scale is another important psychometric property, which is the prerequisite of comparison across repeated assessments. Researchers are encouraged to further explore the longitudinal invariance in the future.

## Conclusion

Collectively, the current study developed and provided initial support for the psychometric properties of the CSMS-CC among two samples of coaches from China. Results of the study indicated that the 10-item three-factor CSMS-CC could be used to measure the CSM of Chinese coaches. This study provides a reliable and valid tool for future research on CSM among Chinese coaches and makes it possible to explore the factors that may influence the CSM of Chinese coaches and its consequences that related to the career development and performance of coaches. This study contributes to the literatures in the field of coach career development and education.

## Data availability statement

The raw data supporting the conclusions of this article will be made available by the authors, without undue reservation.

## Ethics statement

The studies involving human participants were reviewed and approved by the Ethics Committee of Chongqing University. The participants provided their written informed consent to participate in this study.

## Author contributions

CZ contributed to conceptualization, formal analysis, investigation, data curation, writing—original draft preparation, and project administration. J-DL contributed to methodology, validation, writing—review and editing, supervision, and editing. Both authors had read and approved the final manuscript.
